# Family planning science and practice lessons from the 2018 International Conference on Family Planning

**DOI:** 10.12688/gatesopenres.13130.2

**Published:** 2021-03-02

**Authors:** Jean Christophe Rusatira, Claire Silberg, Alexandria Mickler, Carolina Salmeron, Jean Olivier Twahirwa Rwema, Maia Johnstone, Michelle Martinez, Jose G. Rimon, Linnea Zimmerman

**Affiliations:** 1 Bill & Melinda Gates Institute for Population and Reproductive Health, Department of Population, Family and Reproductive Health, Johns Hopkins Bloomberg School of Public Health, Baltimore, MD, 21205, USA; 2 Department of Population, Family and Reproductive Health, Johns Hopkins Bloomberg School of Public Health, Baltimore, MD, 21205, USA; 3 Department of Epidemiology, Johns Hopkins Bloomberg School of Public Health, Baltimore, MD, 21205, USA

**Keywords:** Family planning, return on investment, women empowerment, reproductive rights, reproductive health, gender empowerment, contraceptive technology

## Abstract

**Background**

Since 2009, the International Conference on Family Planning (ICFP) has served as an opportunity for the global reproductive health community to share FP advances and practice lessons in the areas of research, programming, and advocacy. The purpose of this paper was to synthesize the key results and findings presented by members of the FP community at the 2018 ICFP Conference.

**Methods**

More than 700 abstracts from all 15 conference tracks were reviewed and 64 abstracts total were selected for this paper based on the novelty and urgency of the findings. The content analysis of conference abstracts were grouped into six final thematic areas.

**Results**

1 **)** *Investing in family planning for a lifetime of returns*. FP continues to face a shortage of funding. Domestically based and locally owned funding models provide alternative financing solutions. 2) *Addressing inequities in family planning for key populations.* Various populations still face challenges in accessing FP. Youth-inclusive and user-centered programming show promise in addressing such challenges. 3) *Reproductive justice, *Unsafe abortions tend to be more common among younger, poor, uneducated and rural women. Legislation is still needed to facilitate a culture of safe abortions. 4) *Couple dynamics and decision-making. *Couples who share equitable responsibility in decision-making processes are more likely to use contraceptives; couple disagreement influences women’s decisions to covertly use FP. 5) *Male involvement in  programming. *Male champions can successfully promote uptake of FP. Gender-transformative programming promotes gender equity and impacts behavior change. 6) *Breakthroughs in novel contraceptives and systems improvement in family planning.* Recent advances include user-centered contraceptive technologies that allow for self-administration and information systems which optimize supply chain management.

**Conclusion**

The research, advocacy, and programmatic abstracts at ICFP 2018 highlighted research advances, showcased implementation science wins, and provided evidence of critical knowledge gaps in global FP access and use

## Disclaimer

The views expressed in this article are those of the author(s). Publication in Gates Open Research does not imply endorsement by the Gates Foundation.

## Introduction

The family planning (FP) community acknowledges that access to safe, high quality, voluntary family planning is a human right. However, the majority of girls and women, particularly in developing countries, continue to have limited and inequitable access to sexual and reproductive health rights, information, and services, including FP ^1^. Although more than 500 million couples in developing countries use FP, the United Nations estimates that by 2030, nearly 200 million women seeking to delay or avoid having a birth will have an unmet need for modern contraception ^2^. This demand will likely continue to grow as record numbers of young people enter the prime reproductive ages in the decades to come. It is thus essential that the family planning community identifies high impact approaches to address the major barriers and gaps affecting equitable access to quality family planning.

Since its inception in 2009, the International Conference on Family Planning (ICFP) has served as a strategic inflection point for the FP and reproductive health community worldwide. ICFP serves as an international forum for scientific and programmatic exchange that enables the sharing of available findings and the identification of knowledge gaps, in addition to facilitating the use of new knowledge to transform policy. At the London Summit in 2012, the global FP community set an aspirational goal to enable 120 million more women and girls to access voluntary quality FP by 2020, and the FP community broadened that goal to include universal access to reproductive health care and services by 2030 ^3, 4^. The ICFP has been an important, collaborative effort in the buildup to establishing that goal, raising visibility, creating momentum around FP, and leading to concrete changes in policy and programs.

The 2018 ICFP, held in Kigali, Rwanda, was centered on the overarching theme, “Investing for a Lifetime of Returns”. This theme was chosen because of the essential role of FP for the realization of all 17 Sustainable Development Goals (SDGs) and spoke to the various returns that investments in FP provides — from better sexual and reproductive health outcomes and improvements in maternal and child health, to education and women’s empowerment, to long-term environmental benefits and socio-economic growth ^5^. Over 700 oral presentations were featured at the conference and covered FP advocacy wins, services developments, and research. Oral presentations were grouped into the following conference tracks: 1) Returns on investment in family planning and the demographic dividend; 2) Policy, financing, and accountability; 3) Demand generation and social and behavior change; 4) Fertility intention and family planning; 5) Reproductive rights and gender empowerment; 6) Improving quality of care, 7) Expanding access to family planning; 8) Advances in contraceptive technology and contraceptive commodity security; 9) Integration of family planning into health and development programs; 10) Sexual and reproductive health and rights among youth and adolescents; 11) Men and family planning; 12) Family planning and reproductive health in humanitarian settings; 13) Faith and family planning; 14) Urbanization and reproductive health; 15) Advances in monitoring and evaluation methods. This paper summarizes the highlights of the scientific program and identifies key findings presented during the oral sessions in the fields of research, programming, and advocacy in order to inform future work in these fields.

The findings summarized in this paper are from 64 abstracts from individual and preformed panel submissions accepted for oral presentations at ICFP 2018. Each co-author of this paper reviewed abstracts from up to three conference tracks based on their expertise and provided summaries from these tracks, organized by emerging key themes. The final abstracts were selected for inclusion in this paper based on the novelty of the findings and contribution to the FP field. These summaries were incorporated to develop the final draft of the paper. 

## Lessons from ICFP 2018

### Investing in family planning for a lifetime of returns

Measuring the returns on investments in FP is crucial for continued funding and support for FP programs. The business cases for FP presented at ICFP demonstrated the ways in which cost-effective FP programming may save money in the short-term and long-term at the individual, community, donor, and national levels. Willcox and colleagues developed a model based on 47 county referral hospitals in Kenya, which demonstrated that for every dollar invested in training and equipment for implant removal services, a future return of USD $1.62 would be accrued from the economic benefits of continued implants uptake ^6^. Costing data presented by Tumusiime and colleagues found that in Senegal and Uganda, the total costs—including direct medical costs (i.e. provider time, supplies, drugs), costs of self-injection training (based on a one-page instruction sheet scenario), and direct non-medical costs (i.e. client travel and time costs)—are significantly lower for the self-injection of depot medroxyprogesterone acetate administered subcutaneously (DMPA-SC) as opposed to provider-administered injectables ^7^. In Nigeria, Adedeji and colleagues found that for every $1 invested in high-impact intervention-focused FP programs, an estimated $1.40 may be saved on maternal and newborn care, and another $4 could be saved on treating complications from unplanned pregnancies ^8^. While self-administered DMPA-SC may provide a cost-effective approach to improving access to long-acting reversible contraceptive (LARC) methods, a study conducted in Rwanda identified LARCs to be more cost-effective than non-LARC methods post-partum, with a savings of $31.42 per pregnancy averted for two years following birth, and additional cost savings expected over longer time frames ^9^.

FP may also be a catalyst for the demographic transition and an opportunity to realize the benefits of the demographic dividend. The demographic dividend describes the changes in the population age structure caused by reductions in population-level fertility and mortality rates. These structural population changes result in a large working-age population and a smaller number of youth dependents ^10^. With the correct set of political, economic, educational, and employment policies and opportunities, countries characterized by this population age structure have the potential to take advantage of the large working age population to bolster socio-economic development and create generational wealth ^11^. Furthermore, this demographic transition may help countries achieve SDG targets. Modeling has shown that FP investments can positively affect SDGs across several sectors including health, governance, economic growth, agriculture, and education ^12, 13^. Despite improvements in FP funding and financing, expanded financial investments in FP are still needed throughout much of sub-Saharan Africa in order to successfully reach the FP targets necessary for countries to reap their demographic dividend potential ^14, 15^.

Strategies to sustain FP advances include long-term financing for FP, particularly the transition from donor-dependent financing to locally owned initiatives. Donor funding to support FP continues to fall short of the amount needed to address the unmet need of family planning globally and the extent of this gap varies significantly across countries and regions ^16^. To mitigate the impact of this shortage in donor funding, it is critical for countries to plan for shifts in financing options, including the procurement of finances for subsidized commodities. Locally owned community-based health insurance (CBHI) schemes, characterized by voluntarily pooled funds, may be a promising option in order to sustain FP financing ^17^. Research on CBHI schemes from sub-Saharan Africa showed positive effects on healthcare utilization and FP uptake. In Ethiopia, Pathfinder International found that women who were enrolled in a CBHI scheme were 1.3 times more likely to practice modern FP than those who were not enrolled ^18^. Since 2014, the Ethiopian government has slowly shifted away from donor-dependence and has launched and expanded the number of CBHI and social health insurance (SHI) programs in more than one-third of districts. Based on current projections, by 2025, the number of modern contraceptive users in Ethiopia will have doubled from 6 million to 12 million, and the private sector will account for 40% of them ^19^.

Data gleaned from nationally representative datasets showed a similar global pattern in factors associated with FP utilization. Findings from the Ethiopia (2016), Kenya (2014), Nigeria (2013), and Philippines (2013) Demographic Health Surveys (DHS), as well as Indonesia’s 2015 Susenas survey, revealed trends in the number of insured women and the modern contraceptive prevalence rate (mCPR); specifically, the ratio of mCPR between insured versus uninsured individuals was greatest among women of the lowest socioeconomic status (SES) in the Philippines, Kenya, Indonesia, and Ethiopia ^20– 23^. Insurance coverage was shown to be directly associated with FP utilization. These findings signify the importance of comprehensive health insurance for FP access, particularly amongst marginalized groups ^24^. Another important finding related to FP access and insurance showed how national health priorities supersede FP access. While FP is often included under universal health coverage (UHC) schemes, the inclusion of FP is often not operationalized or realized ^25^. Data from 22 priority FP2020 countries showed that the challenges to comprehensive UHC include government prioritization of less cost-effective yet urgent curative services, instead of preventive care or primary services ^26^. 

Additionally, research on health financing highlighted opportunities for new financing models and insurance schemes. In Tanzania, the United Nations Fund for Population Activities (UNFPA) and DKT International implemented an innovative micro-insurance scheme for urban youth and adolescents, which demonstrated high uptake in just one year of initiation. This program, “iPlan”, required a nominal annual fee of $10, after which an individual received comprehensive sexual and reproductive health (SRH) services including contraceptive counseling and commodities for one year ^27^. Similarly, researchers found that the Public-Private Partnership Health Posts model in Rwanda was a cost-effective and viable solution for individuals living more than 60 minutes away from health facilities ^28^. The social franchising model created by the Family Health Guidance Association of Ethiopia (FGAE) was also shown to be a cost-effective model as compared to static clinics. When compared to the FGAE-owned static clinics, the cost per Couple Years of Protection (CYP), (an indicator used to estimate protection from pregnancy by family planning/contraceptive methods during a one-year period) ^29^ was significantly less expensive. CYP provided through the FGAE social franchise model was estimated to be between USD $0.73-$1.77, compared to USD $25.61-37.35 per CYP provided at the FGAE-owned static clinics ^30^.

### Addressing inequities in family planning for adolescents, youth, and key populations

Inequities in access to FP exist across women from different socio-economic groups, age cohorts, health statuses, and physical abilities. Compared to women of other reproductive ages, adolescent girls and young women (AGYW) have specific FP and sexual and reproductive health needs, including low contraceptive uptake, high risk of unintended pregnancies and unsafe abortions, high risk of sexually transmitted infections, and a greater risk of acquiring HIV ^31, 32^.

Involving youth in advocacy and programming efforts was shown to be critical in order to ensure that their unique FP needs are met. Reproductive Health Uganda developed an innovative program to support young people in realizing their right to hold state-actors accountable for improving access to youth-friendly health services. The initiative led to the successful allocation of county-level funds for youth-friendly services in all sectors and created a network of youth advocates for FP programming ^33^. In Kenya, the Network for Adolescents and Youth of Africa developed a holistic advocacy network in Kisii County that led to the allocation of KES 7,000,000 (USD 68,000) to contraceptive procurement and FP services in the financial year 2016/2017, the first time a line item for FP was included in the county budget ^34^.

FP programs for youth with hearing and speech impairments included a sexual health education program for adolescents in Vietnam and a social media literacy program integrating SRH and FP information exchange in Burkina Faso ^35, 36^. In Egypt, Love Matters Arabic Project was launched to engage young people on SRH issues, dispel myths and taboos, and improve access to accurate and reliable SRH and FP information ^37^. Some researchers maintain that to attract youth and gain their trust, programming must include a pleasure component and tie this information to healthy sexual behaviors and practices ^29, 38^. This hypothesis needs further exploration in future research and programming.

Other key populations highlighted during the conference included youth living in conflict zones, people living with HIV, women with disabilities, female sex workers, people who use drugs, individuals with a low socioeconomic status, and individuals who do not identify as heterosexual ^39, 40^. A nationally-representative survey from Ethiopia found that more than 95% of women living with a mental, physical, or visual disability face obstacles in physically accessing health facilities and are less likely to have access to FP information ^38^. Furthermore, this sub-population may be more likely to face discrimination by healthcare providers. These barriers to FP services and knowledge may have direct consequences on health outcomes. For example, among women with disabilities who have ever had a pregnancy, more than 85% reported that the pregnancies were unintended ^41^.

Studies from conflict zones in Afghanistan, Cameroon, Liberia, Sierra Leone, and Yemen showed that girls who marry before the age of 18 have lower rates of FP use, less intention to use in the future, and a significantly higher risk of unintended pregnancy, compared to married women 18 years of age and older ^42^. Among Somali refugee girls aged 10–19 and living in Ethiopia, nearly 75% of girls were aware of how to become pregnant, but fewer were aware of the risks associated with inadequate birth spacing. Despite nearly one in five girls having already given birth, 40% of participants remained unaware of methods to avoid pregnancy ^43^.

People living with HIV may also have trouble accessing comprehensive FP services. A study from Uganda found that unmarried women with an HIV-positive status and women of high parity were significantly less likely to use FP post-partum ^44^. Women who take antiretroviral therapy have desires to bear children, learn about contraception, and receive information on methods to prevent mother-to-child transmission of HIV ^45^. To this end, it is important that programs recognize this population’s unique desires and needs. A program in London demonstrated the promise of service integration to improve access to FP for women living with HIV; Mabonga and colleagues found a 50% increase in LARC use after the integration of FP and HIV services in a postnatal contraception clinic in London ^46^. Integrating HIV and FP services into one convenient location helps promote healthy SRH and child health outcomes, while also easing client burden associated with traveling between different clinics.

### Reproductive justice: Abortion care, family planning, and women’s wellbeing

Unsafe abortions have emerged as one of the key neglected public health problems, accounting for more than 1 in 10 maternal-related deaths worldwide ^47^. Accordingly, abstracts discussing safe abortion access and FP were cross-cutting through the conference’s tracks. Research on unsafe abortions underscored the determinants of abortion practices as well as inequities in the accessibility of safe abortion services. For example, in both Nigeria and Rwanda, younger, uneducated women in rural areas are more likely to seek out and use abortion services. However, due to restrictive abortion laws, these abortions are often unsafe, which poses not only health challenges but legal challenges as well ^48^. In 2012, 24% of all incarcerated women in Rwanda were imprisoned for participating in clandestine, illegal abortions ^49^. Access to safe abortion services is a critical component of comprehensive SRH yet continues to be heavily restricted in many parts of the world. Several authors called for targeted advocacy for legal provisions to ensure the availability of safe abortion services ^50, 51^. Amendments to national laws, increased and expanded training of providers, and improved access to medical abortions were highlighted as priorities for policymakers ^24, 52^. Furthermore, emphasis was placed on the recognition of social disparities and inequities in abortion prevalence and access ^45^.

Analyses of post-abortion care (PAC) programs for women in humanitarian settings in DRC and Yemen found that providers may effectively shift from unsafe practices of dilation and curettage (D&C) to manual vacuum aspiration and medical treatment with misoprostol. Over a period of 5 years, the percentage of PAC clients requiring evacuation who received D&C as treatment was reduced from of 18.6% to 2.0% in DRC and from 25% to 2.8% in Yemen ^53^.

Expanding access to safe abortion services can also directly increase women’s access to FP. Research from Kenya found that, regardless of pregnancy intentions, over 70% of women who attended PAC initiated contraceptives during their PAC visit ^54^. Analyses of post-abortion family planning (PAFP) service delivery across two states in India also revealed that 28% of women adopted a contraceptive method within two months after their abortion ^55^. Another study from Kenya found that women’s PAFP method varied based on the type of abortion the woman experienced. While women who had undergone surgical abortions were more likely to choose intrauterine devices or other LARC methods, women who had medical abortions were more likely to choose implants. While this may be due to the fact that IUDs can be inserted following a surgical abortion but not following a medical abortion, further research is necessary to ensure women receive the FP method that best suits their needs, preferences, and fertility desires ^56^. Insights into context-specific ideals of family size as well as abortion care-seeking behaviors are important in understanding how to improve future PAFP service delivery and increase contraceptive use ^51^.

### Couple dynamics and family planning decision-making

Research on women’s covert use of FP underscored the ethical tensions between supporting and validating women’s ability to exercise reproductive autonomy without disclosure to a partner while also striving to engage male partners in reproductive health decisions ^57^. Research revealed that a woman’s decision to covertly use FP may be linked to discordant partner views on childbearing and fertility desires ^58^. One study found that when men expressed beliefs that contraception is “women’s business”, women were more likely to engage in covert use and not disclose their FP decisions to their partners ^53^. However, women who use FP covertly often struggle with the cost of contraceptives and worry about concealing FP from their partners ^53^. Power dynamics continue to influence FP use, even when women choose to use FP methods covertly.

Couple power dynamics and household decision-making also influences FP utilization. Easterlina and colleagues found that 75% of women in West Pokot, Kenya, identified their husband or partner as the biggest barrier to voluntary FP use ^59^. In the Afar region of Ethiopia, 58.8% of women reported not having the freedom to make independent fertility decisions ^60^. Conversely, researchers have found that the odds of using modern contraception increases significantly when couples make decisions together ^61^. Couples who reported shared decision-making on everyday life choices (e.g. financial decisions) in Ibadan, Nigeria, were more likely to report using FP than couples in which decisions were made solely by the husband ^62^. Other factors which have been found to influence FP uptake include the educational status of couple dyads, couple’s knowledge of reproductive health and rights, women’s economic security and involvement in microcredit schemes, and gender equitable household dynamics ^63, 64^.

### Male involvement in family planning programming

Considering men’s influence on FP decisions, involving male partners in FP programming is essential to meeting FP goals globally. Males have a desire to learn about FP and contraception but often have limited or inaccurate information which fuels false beliefs and myths. In Uganda, when men were asked why they do not allow their wives to use modern FP methods, participants expressed fears that their wives were likely to become promiscuous if they began using contraception. The researchers also found that male participants’ beliefs about FP were often inaccurate, inconsistent, or grounded in gendered stereotypes, fueling fears about wives’ promiscuity ^65^. Similarly, research from Kenya showed that 50% of men in Western Kenya lack accurate knowledge on the possible benefits of healthy timing and spacing of pregnancies ^55^. In Nepal, men’s limited understanding of contraceptives were shown also to impact their partner’s uptake of IUDs ^66^.

Research revealed the potential of male champions and advocacy networks in changing social norms, educating male peers, and creating a culture receptive and open to family planning discussions. In Uttar Pradesh, India, a community-based information diffusion strategy was used to dispel FP myths and misconceptions and provide comprehensive information on non-scalpel vasectomy. To accommodate the diverse lives of men living in informal settlements, men were engaged by their peers at traditional male gathering points at convenient times, such as evening meetings for rickshaw pullers ^67^. In Zamboanga City, Philippines, a packaged community-based learning program, EL HOMBRE, used a peer-to-peer information dissemination technique to share information related to FP, family matters, and family planning ^68^. Similarly, a male champions program was rolled out successfully in Western Kenya, where 50 male champions held sensitization forums once a month to encourage discussions on healthy timing and spacing of pregnancies ^55^. In Benin, USAID/ANCRE implemented a “men as advocates” intervention that included counseling male spouses on FP when their partners left the maternity ward and creating groups of “committed men” to sensitize male peers. Over the course of a year, post-partum FP counseling for males increased by more than 100% across 47 health facilities ^69^.

Couple-based approaches to behavioral change and FP uptake also show promise. Project Concern International implemented a social and behavioral change program that used couples as community change agents to address restrictive social norms and SRH myths, improve couple communication strategies, and aid couples in the development of their FP and fertility goals ^70^. The Emanzi program in Uganda also showed a positive changes in equitable gender norms, a rise in shared decision-making in the household, and a significant increase in FP uptake ^71^.

Gender-transformative programming is grounded in the notion that changes in gendered norms, beliefs, and behaviors lead to positive health outcomes. Landmark gender-transformative programs included the Bandebereho intervention in Rwanda, which consisted of 15-week group education meetings for more than 4,000 young adult men and women and 1,700 expectant and new fathers and couples. When compared to the control group, findings showed an increase in the proportion of young people who had sought SRH services, as well as changes in positive gender norms and increases in shared decision-making ^72^. The GroupUp Smart education curriculum in Rwanda targeted prepubescent male and female adolescents and their parents. The program found that adolescent boys’ awareness of preventing pregnancy increased from 65% to 81% and their knowledge of reproductive health significantly increased. Compared to pre-intervention, adolescent boys experienced significant increases in gender equity scores, pointing to the notion that SRH education which includes a gender component may be more beneficial than SRH education alone, particularly when introduced earlier in life ^73^.

### Breakthroughs in novel contraceptives and systems improvement in family planning

Research advances in contraceptive technology highlighted the importance of beginning with the end-user in mind. In Nigeria and India, initial acceptability research of a microneedle contraceptive patch (MNP) explored client perceptions of the method and quantified desired MNP attributes. Across both contexts, prospective users liked the potential for self-application and both providers and clients found the method to be easily used. Researchers also wanted to identify user preferences for other attributes, including the method’s effect on menstruation, duration of effectiveness, placement location, pain, and the potential for skin reactions at the application site ^74^. These findings underscored high overall acceptability of microneedles as a novel delivery method, yet also emphasized the importance of reducing side effects associated with existing contraceptive methods.

Use of the levonorgestrel intrauterine system (LNG-IUS) has risen rapidly in high-income countries and is one of the most effective forms of contraception available. However, the cost of the method is typically a barrier to clients in low-income countries. Research by Marie Stopes International Nigeria and FHI360 piloted the introduction of an affordable version of the LNG-IUS at multiple service delivery points and found that users, providers, and key opinion leaders were receptive and enthusiastic about the method. Many clients also reported reduced menstrual bleeding as a key non-contraceptive benefit of the method. This research also suggested that a multi-stakeholder approach, including coordinated demand-generation activities, may be important in order to advance the scale-up of LNG-IUS in Nigeria and in other similar contexts ^75^.

Improved access to subdermal implants and other long-acting methods like IUDs have raised concerns on whether women can access timely removal services on-demand. Data from pilot studies examining the subdermal implant removal tool, RemovAid, suggested that this novel device is safe to proceed to larger studies, and with it, physicians can safely remove one-rod implants and minimize the removal time to just under seven minutes ^76^. Furthermore, initial acceptability research revealed that a novel postpartum IUD inserter would be attractive in India due to high unmet need and a lack of trained providers ^77^. These products would not require additional supplies, aside from what it’s packaged with, and demonstrated high client and provider satisfaction.

Novel approaches to service delivery and contraceptive commodity procurement included the development of an “informed push” model, which would change the public health sector’s reporting system to allow for consolidated transport routes and combined supply delivery. Rather than following a typical model where an individual health facility is responsible for FP commodity reporting, product requisition, and pick-up, this model relied on health “zone staff” to optimize transport routes and report on stockouts and product consumption. By consolidating FP commodities alongside other health products and optimizing transit routes, the study demonstrated a substantial reduction in the incidence of stockouts and a decline in transit costs ^78^. In India, an application developed by the Ministry of Health and Family Welfare also seeks to collect consumption data, forecast demand, and track commodity distribution. While still in the formative stage, individual states have demonstrated an interest in customization of the app per state to allow the government to improve commodity distribution and transfers by tracking “live” data ^79^.

Lastly, algorithm-based fertility apps, such as the Dynamic Optimal Timing application, demonstrated a typical-use failure rate that was comparable to or better than other user-initiated methods, including fertility-awareness based methods. This method delivered consistently correct information to women about their daily fertility status, which suggests that the app could allow women to self-manage fertile days to avoid pregnancy ^80^.

## Discussion

The 2018 ICFP scientific program underscored new advances in family planning research, programs, and advocacy work, that have important practical and policy implications. Short- and long-term benefits of FP investments were highlighted, from increased empowerment at both the individual and couple levels to reduced maternal mortality and improved population health. Nevertheless, achieving these dividends as a result of FP investments continues to be thwarted by insufficient funding, limited contraceptive choices, and persistent inequality in accessing FP programs and services.

The growing reproductive-age population, particularly in developing countries, and the increasing demand for FP requires innovative financing initiatives to meet the demand and ensure resilient health systems. Community-based health insurance schemes and public-private partnerships between the Ministries of Health and local businesses are promising solutions to ensure that all girls and women with unmet need can access and utilize FP. Future research should focus on scaling cost-effective, self-administered technologies.

While progress is being made globally on improving access to contraceptive services, urgent actions are required to address the FP needs of specific subpopulations that lag behind. These populations include AGYW, female sex workers, women and girls with disabilities, women living with HIV, and populations living in conflict-afflicted regions as well as other humanitarian settings. Research focusing on such populations is becoming increasingly highlighted at ICFP but remains very limited compared to research and program efforts focused on other populations. Future research should explore the needs of such unique sub-populations and evaluate interventions and programs that may successfully be scaled to address the FP needs of these marginalized groups. Gender and social norms continue to play a key barrier in FP demand generation. Further research is needed to evaluate the effectiveness of gender transformative programs that aim to address gender norms that perpetuate social and health inequalities. Empowerment efforts need to continue to engage men as partners while considering women’s autonomy in FP decisions, and ensure that context-specific couple dynamics and social norms are integrated into programming.

Despite achievements and advances in FP access and utilization, the abortion space still lags behind. Unsafe abortions and abortion-related fatalities remain a neglected and preventable public health problem. Current and future advocacy efforts should focus on the legal provision of abortion care to ensure the availability of safe, decriminalized abortion services. Such efforts should be undertaken in parallel with expanded training for providers, while utilizing the opportunities to integrate FP methods in post-abortion care. To further understand PAC, future research is needed to determine what influences a woman’s decision to use contraceptives post-abortion and the specific method choice selected, and why.

Continued improvements in information systems have allowed for the rapid reporting of inventories, consolidated transport routes, and combined supply delivery. Such systems present an opportunity to address supply chain challenges and prevent stock-outs from the sub-national to the national levels. Artificial intelligence and algorithm-based applications present opportunities for FP information access through mobile user technologies. Allowing such systems to communicate with the supply chain may allow women to better access their contraceptive method of choice and allow couples to achieve their desired family size.

Implementation science research should also focus on understanding the key drivers that affect the uptake of research findings. This research can be used to inform evidence dissemination and utilization by policymakers and other decisionmakers at the local and national levels. FP is not only a social justice issue, but a smart investment for individuals and communities. Ensuring that local leaders and policymakers properly understand these two rationales for FP could be key to success for the global community and may lead to more prosperous and resilient communities. Over the last few years, the concept of the demographic dividend has provided a broader ground for advocates to support FP efforts. The economic theory of the demographic dividend tends to resonate well with policymakers and peoples from various religious backgrounds, including religious leaders. Nevertheless, challenges remain for the human-rights rationale to be as widely accepted as the economic theory.

## Conclusion

ICFP 2018 generated rich evidence on successes achieved in recent years and highlighted continued gaps in research, implementation and advocacy. Science and practice lessons demonstrated the need for a multi-sectoral, interdisciplinary approach among FP stakeholders in order to inform new actions to attain the 2030 universal access goal. The universal access goal presents an opportunity for the world to close the gap in FP inequities between individuals of different socioeconomic backgrounds and attain shared prosperity across communities. Investing in FP paves the path for generational wealth and a range of health returns. Addressing FP advocacy, services, and research challenges and continuously sharing lessons learned and best practices through platforms such as ICFP will be essential for countries to accelerate progress towards the universal access goal and ultimately, meet the needs of all women and girls.

## Data availability

All data underlying the results are available as part of the article and no additional source data are required. ****
