# Supplementary material for: Family planning science and practice lessons from the 2018 International Conference on Family Planning
Source: Gates Open Res. 2021 Mar 2;4:43. Originally published 2020 Apr 27. [Version 2] doi: 10.12688/gatesopenres.13130.2 (PMC7374012; doi:10.12688/gatesopenres.13130.2)
Supplement: Peer review history

# Peer review: Reviewer response for version 1

**DOI:** 10.21956/gatesopenres.14317.r29010

**Published:** 2020-07-27

**Authors:** Gillian Mckay

**Affiliations:** 1 Department of Global Health and Development, London School of Hygiene and Tropical Medicine, London, UK

Thank you for the opportunity to review this Open Letter that summarizes the key findings from the 2018 International Conference on Family Planning that was held in Kigali, Rwanda. The letter brings together 6 overarching themes drawing across the 15 tracks of the conference, to provide a concise yet comprehensive overview of the state of the evidence in family planning programming, advocacy and research.

 I have some minor recommendations to improve the paper:

 Methods:

- It was not clear to me how many panels there were at the conference. Were the 65 individual and preformed abstracts the sum total of the 700+ oral presentations made? If this was a sub-section, how were these abstracts chosen for inclusion?
- How did the process of thematic grouping of the 15 tracks into 6 themes take place?   
- Include a line in the abstract around the methods.

General Comments:

- Quite technical language is used from time to time, which may be inaccessible to those outside of the FP space. e.g. Community Based Health Insurance & Couple Years of Protection: these terms could be better explained in the text or in a footnote.
- There are too many acronyms, many of which are only used once, therefore could likely be removed to make the paper easier to read.
- Some light copy-editing is needed for grammatical errors.

Does the article adequately reference differing views and opinions?

Yes

Are all factual statements correct, and are statements and arguments made adequately supported by citations?

Yes

Is the Open Letter written in accessible language?

Partly

Where applicable, are recommendations and next steps explained clearly for others to follow?

Yes

Is the rationale for the Open Letter provided in sufficient detail?

Yes

Reviewer Expertise:

Reproductive health in humanitarian crises, with a focus on outbreaks of infectious disease.

I confirm that I have read this submission and believe that I have an appropriate level of expertise to confirm that it is of an acceptable scientific standard.

# Response

**Published:** 2020-10-22

**Authors:** Jean Christophe Rusatira

**Affiliations:** Johns Hopkins School of Public Health, USA

Dear Gillian,

Thank you for your thorough review of our paper. Please find below our responses to your comments in *italic* and marked with bullets.

Sincerely,

Jean Christophe Rusatira

Methods:

It was not clear to me how many panels there were at the conference. Were the 65 individual and preformed abstracts the sum total of the 700+ oral presentations made? If this was a sub-section, how were these abstracts chosen for inclusion?

- *At ICFP 2018, there were 700+ oral presentations presented, submitted as both individual and performed abstracts. Each abstract is counted as one oral presentation. All abstracts were reviewed for the novelty of their findings and 64 abstracts were selected for the final paper. We clarified this in the Introduction and Abstract.*

 

How did the process of thematic grouping of the 15 tracks into 6 themes take place? 

- *The thematic groupings were based on key findings from the selected abstracts and major thematic areas highlighted in these findings. The 15 tracks were from the abstract submissions and guided the review process, but for the purposes of this paper, new thematic areas were defined based on the main findings from the abstracts.*

 

Include a line in the abstract around the methods.

- *The abstract has been revised and this comment has been addressed.*

General Comments:

Quite technical language is used from time to time, which may be inaccessible to those outside of the FP space. e.g. Community Based Health Insurance & Couple Years of Protection: these terms could be better explained in the text or in a footnote.

- *Thank you for your comment. We have addressed this by explaining CYP and CBHI directly in the text of the paper.*

 

There are too many acronyms, many of which are only used once, therefore could likely be removed to make the paper easier to read.

- *We agree with this comment and have removed all acronyms that only occur once in the paper. We have kept acronyms that are used more than once.*

 

Some light copy-editing is needed for grammatical errors.

- *We have made editorial copy editing to remove grammatical errors.*

# Peer review: Reviewer response for version 1

**DOI:** 10.21956/gatesopenres.14317.r29113

**Published:** 2020-07-21

**Authors:** Nguyen Toan Tran

**Affiliations:** 1 The Australian Centre for Public and Population Health Research, Faculty of Health, University of Technology Sydney, Sydney, NSW, Australia; 2 Faculty of Medicine, University of Geneva, Geneva, Switzerland

Thank you for the opportunity to review this open letter, which offers a remarkably useful summary of the state of family planning programs and research. A few points:

- With regard to the abstract, a line on the open letter objective and methods would help transition between the introduction paragraph and the second one.

- It would be helpful to learn more about why the theme of “Investing for a lifetime of returns” was chosen, taking into account the tensions between the macro level (e.g. economic and environmental) and individual level (e.g. empowerment, rights, and justice, which are just touched upon).

- The second para under “Investing in family planning” feels incomplete without acknowledging that access to quality education and employment opportunities is critical to realize the benefits of the demographic dividend.

- Consider stressing how the conference has embraced and contributed to highlighting the development and humanitarian nexus - as well as safe abortion!

Minor points:

- The frequent use of abbreviations might impede the text flow.
- Slight text editing required (grammar).
- Check references: 2 and 4: UN DESA vs "DESA/Desa, UN". 2: more recent source available? Duplicates 44 & 45?

Does the article adequately reference differing views and opinions?

Partly

Are all factual statements correct, and are statements and arguments made adequately supported by citations?

Partly

Is the Open Letter written in accessible language?

Partly

Where applicable, are recommendations and next steps explained clearly for others to follow?

Yes

Is the rationale for the Open Letter provided in sufficient detail?

Yes

Reviewer Expertise:

Global health with a focus on sexual and reproductive health and rights, including contraception and postpartum family planning, in development and humanitarian settings

I confirm that I have read this submission and believe that I have an appropriate level of expertise to confirm that it is of an acceptable scientific standard.

# Response

**Published:** 2020-10-22

**Authors:** Jean Christophe Rusatira

**Affiliations:** Johns Hopkins School of Public Health, USA

Dear Nguyen,

Thank you for your thorough review of our paper. Please find below our responses to your comments in *italic* and marked with bullets.

Sincerely,

Jean Christophe Rusatira

With regard to the abstract, a line on the open letter objective and methods would help transition between the introduction paragraph and the second one.

- *The abstract has been revised and multiple section breaks have been added to make reading the abstract easier.*

It would be helpful to learn more about why the theme of “Investing for a lifetime of returns” was chosen, taking into account the tensions between the macro level (e.g. economic and environmental) and individual level (e.g. empowerment, rights, and justice, which are just touched upon).

- *We have made changes in the paper to address this comment: this theme was chosen because of the essential role of FP to achieving the 17 Sustainable Development Goals and spoke to the various returns that investments in FP provide — from reproductive health outcomes, to maternal and child health improvements, to empowerment, increases in education, and population-level socioeconomic growth.*

The second para under “Investing in family planning” feels incomplete without acknowledging that access to quality education and employment opportunities is critical to realize the benefits of the demographic dividend. 

- *This was addressed in the new iteration of the paper.*

Consider stressing how the conference has embraced and contributed to highlighting the development and humanitarian nexus - as well as safe abortion!

- * This was addressed in the new iteration of the paper.*

 Minor points:

The frequent use of abbreviations might impede the text flow.

- *We have removed all acronyms that only occur once in the paper. We have kept acronyms that are used more than one time.* 

Slight text editing required (grammar).

- *Editorial copy editing was provided to remove grammatical errors and improve the flow of the paper.* 

Check references: 2 and 4: UN DESA vs "DESA/Desa, UN". 2: more recent source available? Duplicates 44 & 45?

- *This has been addressed in the new iteration of the paper. *

# Peer review: Reviewer response for version 1

**DOI:** 10.21956/gatesopenres.14317.r28799

**Published:** 2020-05-20

**Authors:** Ann Biddlecom

**Affiliations:** 1 Guttmacher Institute, New York City, NY, USA

This open letter concisely summarizes the wide range of scientific inputs to the 2018 International Conference on Family Planning. It serves as a snapshot of the state of current evidence and interest on family planning. What follows are a few suggestions for revision and minor corrections. 

1. It would be useful to take a further step back from the analysis of content to raise the larger debates on framing family planning that can often be in conflict among stakeholders with different objectives and agendas for action (government, donor, advocates): e.g., Demographic Dividend framing with fertility reduction a focus and macro-level benefits emphasized versus a human rights-oriented framing, where individual well-being and attention to inequities and reproductive justice are a central focus. On page 8 this situation is raised but not discussed (“FP is not only a social justice issue, but a smart investment for individuals  and communities.”) 
2. On a related note, could the authors speak to what motivated the thematic framing of the 2018 conference to be “Investing for a Lifetime of Returns”? 
3. At least a nod to job growth and productivity-related policy supports is needed around the demographic dividend explanation (“The demographic transition leads to numerous, subsequent population-level and societal benefits…”). The fertility reductions and age structure shifts are necessary but not sufficient. Education and health investments are required as well as the ability of the economy to productively employ workers. 
4. Abstract: State the evidence and method in one sentence on which the theme-based key points are based (i.e., content analysis of conference abstracts). Also, the general phrase “locally owned models provide alternative financing solutions” is not clear for a general reader, perhaps add an example (such as….) 
5. The abstract has a heavy focus on research alone (“ICFP 2018 highlighted research advances, implementation science wins, and critical knowledge gaps in global FP access and use.”) and yet a substantial part of the program was devoted to utilization (advocacy, policy and program shifts). 
6. (page 6) Clarify if the contrast group is individual decision-making? (“…have been found to be significantly associated with couple’s FP decision-making ^60,61^”) 
7. Explicit attention by the authors (and the conference) to safe abortion is merited as it is a topic and essential intervention often ignored or sidelined in the scientific literature. A helpful contribution of the conference. 

Minor comments:

1. Where possible, minimize the use of acronyms for readability (e.g., AGYW). 
2. Reference 2 is not correct. The statement is about the number of couples in 2030 with unmet need for modern methods (and the 2020 revision is available now for all women, not just married women -- https://www.un.org/en/development/desa/population/theme/family-planning/cp_model.asp), but the reference is a much older publication on population estimates (DESA, UN. United Nations Department of Economic and Social Affairs/Population Division: World Population Prospects: The 2008 Revision. 2009b.) 
3. (page 6) Given the restricted space of an open letter and the number of studies covered, suggest not highlighting the same local study twice (Easterlina and colleagues). 
4. Reference 4 is an official UN publication - the SDGs - and not from the Dept of Social and Economics Affairs (DESA). 
5. References 44 and 45 are duplicates. 
6. Light copy-editing needed (e.g., in abstract “Promising evidence show that…”, “couple discordance…directly influence…”; elsewhere “95% of women living with a mental…faces…).

Does the article adequately reference differing views and opinions?

Partly

Are all factual statements correct, and are statements and arguments made adequately supported by citations?

Partly

Is the Open Letter written in accessible language?

Yes

Where applicable, are recommendations and next steps explained clearly for others to follow?

Yes

Is the rationale for the Open Letter provided in sufficient detail?

Yes

Reviewer Expertise:

Demographic research focused on contraceptive use, abortion, reproductive decisionmaking and adolescent sexual and reproductive health.

I confirm that I have read this submission and believe that I have an appropriate level of expertise to confirm that it is of an acceptable scientific standard.

# Response

**Published:** 2020-10-22

**Authors:** Jean Christophe Rusatira

**Affiliations:** Johns Hopkins School of Public Health, USA

Dear Ann,

Thank you for your thorough review of our paper. Please find below our responses to your comments in *italic* marked with bullets.

Sincerely,

Jean Christophe Rusatira

It would be useful to take a further step back from the analysis of content to raise the larger debates on framing family planning that can often be in conflict among stakeholders with different objectives and agendas for action (government, donor, advocates): e.g., Demographic Dividend framing with fertility reduction a focus and macro-level benefits emphasized versus a human rights-oriented framing, where individual well-being and attention to inequities and reproductive justice are a central focus. On page 8 this situation is raised but not discussed (“FP is not only a social justice issue, but a smart investment for individuals and communities.”)

- *This was addressed in the new version of the paper.*

On a related note, could the authors speak to what motivated the thematic framing of the 2018 conference to be “Investing for a Lifetime of Returns”?

- *Thank you for this comment. We have made changes in the paper to address this comment:* *this theme was chosen because of the essential role of FP to achieving the 17 Sustainable Development Goals and spoke to the various returns that investments in FP provide — from reproductive health outcomes to maternal and child health improvements, to empowerment, increases in education, and population-level socioeconomic growth **.***

At least a nod to job growth and productivity-related policy supports is needed around the demographic dividend explanation (“The demographic transition leads to numerous, subsequent population-level and societal benefits…”). The fertility reductions and age structure shifts are necessary but not sufficient. Education and health investments are required as well as the ability of the economy to productively employ workers.

- *We have revised this section and incorporated information on the investments and political environment necessary to harness the DD.* 

**Abstract**:  State the evidence and method in one sentence on which the theme-based key points are based (i.e., content analysis of conference abstracts). Also, the general phrase “locally owned models provide alternative financing solutions” is not clear for a general reader, perhaps add an example (such as….)

- *We have provided more details to clarify in the Abstract the process of selecting the final themes for the paper.* 

The abstract has a heavy focus on research alone (“ICFP 2018 highlighted research advances, implementation science wins, and critical knowledge gaps in global FP access and use.”) and yet a substantial part of the program was devoted to utilization (advocacy, policy and program shifts).

- *The abstract has been revised considerably and we have attempted to address this comment.* 

(page 6) Clarify if the contrast group is individual decision-making? (“…have been found to be significantly associated with couple’s FP decision-making 60,61”) 

- *            We checked this abstract and changed the wording to provide clarifications.*        

Where possible, minimize the use of acronyms for readability (e.g., AGYW). 

- *We have removed all acronyms that only occur once in the paper. We have kept acronyms that are used more than one time.* 

Reference 2 is not correct. The statement is about the number of couples in 2030 with unmet need for modern methods (and the 2020 revision is available now for all women, not just married women --https://www.un.org/en/development/desa/population/theme/family-planning/cp_model.asp), but the reference is a much older publication on population estimates (DESA, UN. United Nations Department of Economic and Social Affairs/Population Division: World Population Prospects: The 2008 Revision. 2009b.)

- *This has been addressed in the new iteration of the paper. Correct citation: United Nations, Department of Economic and Social Affairs, Population Division (2017). World Family Planning 2017 - Highlights (ST/ESA/SER.A/414).*

(page 6) Given the restricted space of an open letter and the number of studies covered, suggest not highlighting the same local study twice (Easterlina and colleagues).

- *The Easterlina et al. paper was used to augment data on male partners’ lack of education and misinformation related to FP. We have kept the citation but revised the Male Involvement in FP Programming section and provided copyediting to make the section more succinct. * 

Reference 4 is an official UN publication - the SDGs - and not from the Dept of Social and Economics Affairs (DESA).

- *This has been addressed in the new iteration of the paper. Correct citation 4.* *UN (United Nations). 2015. Transforming our world: The 2030 Agenda for Sustainable Development. https://sustainabledevelopment.un.org/post2015/transformingourworld. Accessed 19 August 2020.*

 References 44 and 45 are duplicates. 

- *This has been addressed in the new iteration of the paper.* 

Light copy-editing needed (e.g., in abstract “Promising evidence show that…”, “couple discordance…directly influence…”; elsewhere “95% of women living with a mental…faces…).

- *Editorial copy editing was provided to remove grammatical errors and improve the flow of the paper.*
